# Genome-Wide Identification and Characterisation of *Stress-Associated Protein* Gene Family to Biotic and Abiotic Stresses of Grapevine

**DOI:** 10.3390/pathogens11121426

**Published:** 2022-11-27

**Authors:** Xiaoye Sun, Xue Xia, Xin Guan

**Affiliations:** 1 College of Horticulture and Landscape Architecture, Southwest University, Chongqing 400716, China; 2 Key Laboratory of Horticulture Science for Southern Mountainous Regions, Ministry of Education, Chongqing 400716, China

**Keywords:** grapevine, *SAP* gene family, genome-wide, transcript analysis, biotic stress, abiotic stress

## Abstract

Grapevine is one of the earliest domesticated fruit crops and prized for its table fruits and wine worldwide. However, the concurrence of a number of biotic/abiotic stresses affects their yield. Stress-associated proteins (SAPs) play important roles in response to both biotic and abiotic stresses in plants. Despite the growing number of studies on the genomic organisation of *SAP* gene family in various species, little is known about this family in grapevines (*Vitis vinifera* L.). In this study, a total of 15 genes encoding proteins possessing A20/AN1 zinc-finger were identified based on the analysis of several genomic and proteomic grapevine databases. According to their structural and phylogenetics features, the identified SAPs were classified into three main groups. Results from sequence alignments, phylogenetics, genomics structure and conserved domains indicated that grapevine SAPs are highly and structurally conserved. In order to shed light on their regulatory roles in growth and development, as well as the responses to biotic/abiotic stresses in grapevine, the expression profiles of *SAPs* were examined in publicly available microarray data. Bioinformatics analysis revealed distinct temporal and spatial expression patterns of *SAPs* in various tissues, organs and developmental stages, as well as in response to biotic/abiotic stresses. This study provides insight into the evolution of *SAP* genes in grapevine and may aid in efforts for further functional identification of A20/AN1-type proteins in the signalling cross-talking induced by biotic/abiotic stresses.

## 1. Introduction

Grapevine (*Vitis vinifera L.*) is a perennial fruit crop that is cultivated globally with 8110 varieties [United States Department of Agriculture Foreign Agricultural Service 2022, United Nations FAO, 2019] [1]. In 2020, viticulture occupies an area of 7.3 million ha, with around 85 million tonnes of table grapes, 1.4 million tonnes of dried grapes, and 260 million hL of wine being produced worldwide [International Organization of Vine and Wine, OIV, 2019]. However, in the prosperous grapevine industry, there are increasing challenges from viruses, bacteria, fungi, pests and environmental stresses; for reviews see [2,3,4,5]. Under the frame of agroecology, the strategies for viticulture stress management have been shifted from traditional chemical control to antagonistic anti-microorganism screening and key resistant loci mining for genomics-based breeding [6,7]. The boundary between biotic and abiotic stress-induced plant defence responses are sometimes obscure; for instance, the jasmonic acide (JA) dependent default pathway is triggered by salt stress in grapevine. Their modulation by a parallel pathway activated by pathogen-derived factors has been approved [8]. These biotic/abiotic signals overlap in their upstream events (calcium influx, apoplastic alkalinisation and induction of JA signalling), but differ in their downstream output (stilbene metabolism, different phytoalexin synthesis genes and oxidative burst) [8]. Another example comes from *V. rupestris* affected by O-methylmellein, a conserved pathogenic factor. In this case, O-methylmellein as an amplifier of flg22 shares a specific signature depending on RboH, independent of calcium influx. This means it works neither as a candidate toxin, nor an elicitor activating basal immunity, but as a weapon in interspecies competition by amplifying the basal immune response [9].

Moreover, it is helpful to refer to the classic CODIT (Compartmentalization Of Decay In Trees) and recent findings on the cellular level responses to have a better understanding of host responses to biotic/abiotic stresses [10]. Where the vascular wilt systemically spreads microorganisms or structures such as spores or hyphae, and phytotoxins are mainly restricted to the vessel lumen and cells surrounding vessels, canker is hardly able to spread systemically due to the death of a portion of the vascular cambium that disables the newly formed functional xylem and phloem. Therefore, from the plant side, the resource allocation could be subjected to a trade-off between vessel occlusion and defence reaction in conjunctive living tissues, as well as the signalling transduction cascade within single cells and amongst the symplast through plasmodesmata. The worldwide spread of Grapevine Trunk Disease has been shown to be damaging depending on fluctuating environments as the fungi can be latent in the tissues for years [11]. This may hold true for the other biotic stresses. Therefore, it is interesting to find out about some signalling to diverge the abiotic stresses from the biotic stresses, or branching points upstream or downstream of certain genes.

Among these, stress-associated proteins (SAPs) have attracted much attention. SAPs are a class of zinc-finger proteins containing A20/AN1 domains, which have emerged as important components in plant stress tolerance in the past decade [12,13]. The first identified zinc finger protein was the cytokine-induced protein A20, functioning in the human umbilical vein endothelial cells [14]. This protein is induced by tumour necrosis factor (TNF) and inhibits TNF-induced apoptosis [15]. The other zinc finger domain, AN1, has been characterised for the first time in a ubiquitin-like fusion protein localised in the hemisphere of *Xenopus laevis* eggs and early embryos [16]. As for the C-terminal AN1 domain, it was first found in the protein encoding the hemispheric parent RNA of *Xenopus laevis*. The conserved sequence is CX_2_CX_9-12_CX_1-2_CX_4_CX_2_HX_5_HXC [17]. The AN1 zinc-finger domain is usually found associated with the A20 zinc-finger and such types of proteins are present in many eukaryotes [18].

Though A20/AN1 domains were discovered in animals, their roles have been gradually illuminated in plants. Early studies on *SAPs* have been focused on abiotic stresses at the transcriptional level and gene function. The first plant A20/AN1 protein was cloned from rice (*OsiSAP1*) and showed enhanced expression in response to cold, drought, salt, submersion, heavy metals and injury [19]. In addition, *OsSAP9* (*ZFP177*) and *AtSAP5* were found to be induced by heat, cold and H_2_O_2_, and to play an important role in enhancing temperature stress tolerance [20,21]. Evidence from the *OsiSAP* family as to salinity, osmotic pressure, and hormone and oxidative stresses were reported for a review; see [22,23]. To date, a total of 18 *SAP*s have been isolated from rice, 14 in *Arabidopsis*, and 37 in cotton. *SAPs* have also been identified in other horticulture plant species, such as grapevine, tomato and apple [24,25,26]. 

SAPs interact with many other proteins to execute their functions [27]. There is direct evidence in the cross-talk between biotic and abiotic stress signalling pathways from the first identified *SAP* in a plant that *OsSAP1* plays a key role in basal resistance against pathogen infection [28]. With respect to grapevine, the *SAP* gene family has been studied under abiotic stress. The overexpression of *VaSAP15* rendered the transgenic plants lower in electrolyte leakage and malondialdehyde levels and higher in antioxidant enzyme activity under cold stress [29]. However, the knowledge of *SAP* gene family reactions under biotic stress remains largely unknown. 

In this study, we identified all *VvSAPs* genes and explored their evolutionary relationships, sequence features and duplication events. The tissue-specific expression of grapevine *SAPs* among species under biotic/abiotic stresses were analysed and their potential cross-talking signalling pathways were further discussed. Those will provide references for optimizing the management practices and for mining candidate resistant loci for genomics-based breeding. The signalling pathway induced by *SAPs* encoding the A20/AN1 zinc protein domain will shed light on further study on the biotic and abiotic stresses inducing signalling cross-talking in grapevine.

## 2. Materials and Method

### 2.1. Identification and Sequence Analysis

The nucleotide, protein sequences and GFF files of *V. vinifera* were downloaded from the grapevine genome database (*Vitis_vinifera*-Ensembl Genomes 51). The HMM profile of the SAP DNA-binding domain (PF02037) was downloaded from the Pfam protein families database (http://pfam.janelia.org, accessed on 20 November 2022) and used to survey all grapevine proteins. A local BLASTP search was used to query the full-length amino acid sequences of SAPs from *Arabidopsis* (TAIR-Home Page, arabidopsis.org, accessed on 20 November 2022). To detect conserved domains in each candidate sequence, A20/AN1 zinc-finger motifs were characterised with motif scanning using SMART (http://smart.embl-heidelberg.de/smart/, accessed on 20 November 2022), NCBI-Conserved Domain Database (NCBI Conserved Domain Search (nih.gov, accessed on 20 November 2022)) and InterPro (http://www.ebi.ac.uk/interpro/, accessed on 20 November 2022). Protein sequences containing A20 or AN1 domains were considered members of the candidate *SAPs* gene family in grapevine, and the coding sequences (CDS) were obtained using GFF files by TBtools1.098763 (South China Agricultural University, Guangzhou, China).

The characteristics of VvSAPs, including protein length, isoelectric point (pI), molecular weight (MW) and grand average of hydropathicity (GRAVY), were predicted by the ExPasy website (http://web.expasy.org/protparam/, accessed on 20 November 2022). The subcellular localisation was predicted by Cell-PLoc-2.0 (http://www.csbio.sjtu.edu.cn/bioinf/Cell-PLoc-2/) (accessed on 11 December 2021).

### 2.2. Chromosomal Location and Gene Duplication Analysis

The chromosomal positions of candidates were also obtained from *V. vinifera* genome annotation files (*Vitis_vinifera*-Ensembl Genomes 51). BLAST results and the GFF files of grapevine genomes were inputted into TBtools1.098763 to identify syntenic relationships and used to exhibit the duplication patterns. Physical chromosomal locations were graphically represented by scaling the 8 chromosomes.

### 2.3. Multiple Sequences Alignment and Phylogenetic Analysis

To discover the sequence characteristic, the amino acid sequences of VvSAPs were used for multiple sequence alignments through ClustalX 2.1 with default settings. To compare the evolutionary relationships and identify the subfamilies, the SAPs from grapevine, *Arabidopsis* and rice were used to construct the phylogenetic tree using MEGA7.0 (https://www.megasoftware.net/) (accessed on 12 March 2022) with the neighbour-joining (NJ) method. The bootstrap process was used to detect the tree with 1000 replicates. The phylogenetic tree was then visualised by online software iTol (iTOL: Interactive Tree of Life (embl.de)) (EMBL, Heidelberg, Germany).

### 2.4. Gene Structure, Domain Composition, Conserved Motif and Promoter Regions

The genomic DNA and corresponding CDS sequences of the putative SAPs, including coding sequence distribution, exon–intron boundaries and intron patterns, were analysed with the Gene Structure Display Server (GSDS) (http://gsds.cbi.pku.edu.cn/, accessed on 15 October 2021). Complete protein sequences were submitted to the NCBI-Conserved Domain Database (NCBI Conserved Domain Search (nih.gov, accessed on 15 December 2021)) to find A20/AN1 domain in VvSAPs. Complete amino acid sequences were analysed with MEME (http://meme.nbcr.net/meme/cgi-bin/meme.cgi, accessed on 15 December 2021) to discover conserved motifs in VvSAPs. The DNA sequences (2 kb) upstream of the initiation codon of each candidate gene were downloaded, and cis-regulatory elements were predicted with Plant CARE (http://bioinfor-matics.psb.ugent.be/webtools/plantcare/html/, accessed on 15 December 2021). The combination of gene structure, domain composition, conserved motif, promoter regions and phylogenetic tree were then generated using TBtools1.098763.

### 2.5. Expression Profiles Mining of Grapevine SAPs in Various Tissues, Organs and Developmental Stages

The expression profiles of *VvSAP* genes predicted from an analysis of the grapevine genome releases were analysed in a *V. vinifera* ‘Corvina’ gene expression atlas of different organs at various developmental stages. Microarray data were downloaded from the Gene Expression Omnibus (GEO) under the series entry GSE36128 (https://www.ncbi.nlm.nih.gov/geo/, accessed on 5 March 2022). Expression analyses in response to biotic and abiotic stresses were based on microarray data from Fung et al. (2008), Cramer et al. (2007) and Tattersall et al. (2007) [30,31,32] (series matrix accession numbers GSE31594, GSE31677 and GSE6404), downloaded from GEO datasets. In addition, the expression analysis dataset of biotic stress in casual agents of fungi disease was obtained from the Sequence Reads Archive (SRA) database under the accession number PRJNA692532 [33]. Data were analysed and graphically represented using the mean of Multi Experiment Viewer software. Data obtained from the *V. vinifera* ‘Corvina’ expression atlas were normalised based on the mean expression value of each gene in all tissues/organs analysed, and the other data were calculated as log^2^ fold change in treated and untreated samples.

### 2.6. Plant Material

A suspension cell culture line of *V. rupestris* expressing GFP-*At*TUB6 was used in this study [34]. Cells were cultivated in a liquid medium containing 4.3 g L^−1^ Murashige and Skoog salts (Duchefa, Haarlem, The Netherlands), 30 g L^−1^ sucrose, 200 mg L^−1^ KH_2_PO_4_, 100 mg L^−1^ inositol, 1 mg L^−1^ thiamine and 0.2 mg L^−1^ 2, 4-dichlorophenoxyacetic acid (2, 4-D), which was adjusted to a final pH of 5.8 and supplemented with 30 mg L^−1^ hygromycin B. Cells were subcultured weekly, inoculating 10 mL of stationary cells into 30 mL of fresh medium in 100 mL Erlenmeyer flasks. The cell suspensions were incubated in the dark at 26 °C on an orbital shaker (Shanghai ZhiCheng, Shanghai, China) at 150 rpm [33].

### 2.7. Fungi Isolation, Inoculation and Transcript Analysis

The culture filtrates of *D. seriata* 98.1 and *N. parvum* Bt-67 (isolated from Pyrénées Orientales and Colmar, France, respectively, kindly provided by Prof. Christophe Bertsch, Université de Haute-Alsace, Colmar, France) and *B. dothidea* Sd-18 (isolated from vineyards in Yantai, China) were generated by cultivating 3 plugs of 2×1 cm of the strain grown on solid medium containing 37 g L^−1^ potato dextrose broth (PDB, Solarbio, Beijing, China) with 15 g L^−1^ agar powder (Solarbio, Beijing, China) for 1 week in 250 mL 20 g L^−1^ malt-medium (Bacto malt-extract, Solarbio, Beijing, China) in a 500 mL Erlenmeyer flask in the dark at 28 °C, 200 rpm for 21 days. Further, the *D. seriata* 98.1 culture was filtered with sterilized filter papers to remove the big fungi clusters and the liquid was harvested in an Erlenmeyer flask. After the liquid was vacuum-filtered through a 0.22 µm filter, it was transferred into sterilized 20 mL screw capped vials and stored at −20 °C [33].

An amount of 40 µL of culture filtrate of *D. seriata* 98.1 and *N. parvum* Bt-67 were added to 10 ml *V. rupestris* expressing GFP-*At*TUB6 suspension cells when subcultured. The cells were incubated in the dark in a constant shaker at 26 °C, 200 rpm, for 3 days. As a contrast, a short-term treatment with 2% (*v/v*) of the culture filtrate of *N. parvum* Bt-67 and *B. dothidea* Sd-18 for 24 h was tested. Sterile water (40 µL) was used in the mock-treated control. *V. rupestris* expressing GFP-*At*TUB6 suspension cells were then collected by filtering through a 30 µm pore size nylon net filter in an aseptic vacuum filter system (Millipore, Burlington, MA, USA). The cells were then frozen in liquid nitrogen for RNA extraction. Two biological replicates, each comprising three technical replicates, were conducted for each treatment. RNA extraction and transcriptome sequencing and analyses refer to Zhao et al. (2021) [33].

## 3. Results

### 3.1. Identification of SAP Gene Family in Grapevine

In this study, both Hidden Markov Model (HMM) and BLASTP program were utilized to scan the grapevine database of the 12 X grapevine genome. After the removal of redundant entries, 15 *SAP* genes with A20/AN1 domain were identified, and numbered as *VvSAP1* to *VvSAP15* based on their chromosomal locations according to Vannozzi et al. (2021) [35]. The parameters used to describe the VvSAP proteins are listed in Table 1**,** including deduced protein length, molecular weight, isoelectric point, aliphatic index, and subcellular and chromosome localisations. The deduced length of VvSAPs ranged from 67 (VvSAP14, VvSAP15) to 293 amino acid residues (VvSAP8), whereas the pI ranged from 7.99 (VvSAP5, VvSAP7) to 10.26 (VvSAPs14). The results of the predicted subcellular localisations of these proteins revealed that all 15 proteins may be located in the nucleus, and one also in the chloroplast.

**Figure 1:**
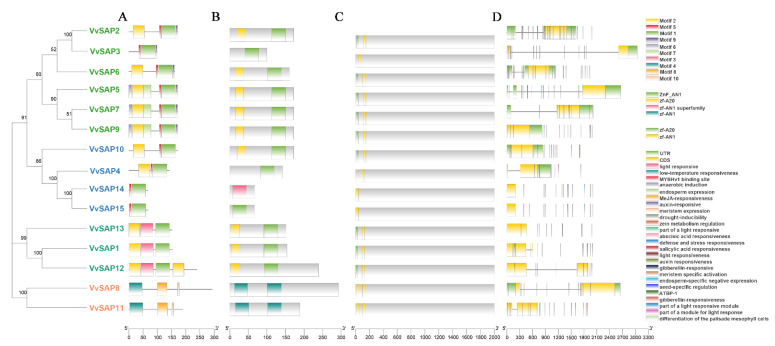
Conserved motif arrangement in grapevine SAP proteins according to the phylogenetic relationship. (**A**) Conserved motif distributions of the VvSAP proteins annotated with the MEME server. Ten motifs are marked by different colours; (**B**) analysis of conserved domains of VvSAP proteins; (**C**) analysis of cis-regulatory elements of VvSAP protein; (**D**) analysis of VvSAP protein coding.

The fifteen candidate genes were subjected to SMART, NCBI-Conserved Domain Database and InterPro analysis for confirmation. All candidates contained the conserved zinc-finger domain AN1, and most also contained the zinc-finger domain A20, allowing validation of all fifteen members of the *SAP* gene family (Figure 1). Among the fifteen SAPs, nine encoded proteins contained both N-terminal A20 and C-terminal AN1 domains, which is the typical configuration for plant SAPs. Four SAPs contained only a single AN1 domain, while two SAPs contained two AN1 domains. Interestingly, no SAPs with only a single A20 domain were found in *V. vinifera*. This is similar to the findings in *Arabidopsis*, cotton and tomato, but not in rice.

### 3.2. Chromosomal Distribution of SAPs 

**Figure 2:**
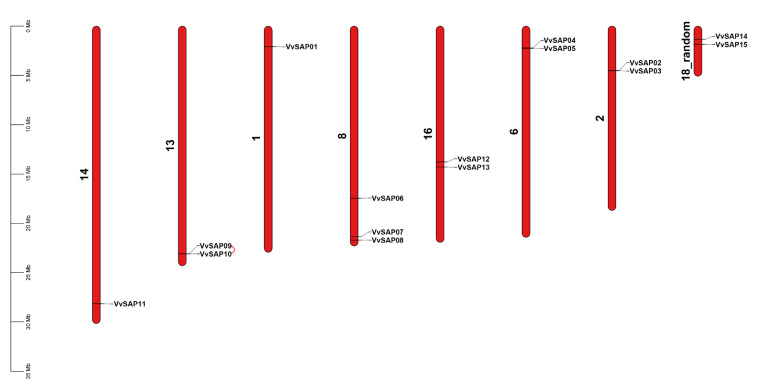
Distribution of the *VvSAP* genes among eight grapevine chromosomes. The scale on the left represents the length of each chromosome displayed in megabase (Mb). Tandemly duplicated genes are coloured with red.

The chromosomal locations of each gene are shown in Figure 2. Three *SAPs* (*VvSAP6, VvSAP7* and *VvSAP8*) are present on chromosome 8, and two *SAPs (VvSAP2* and *VvSAP3, VvSAP4* and *VvSAP5, VvSAP9* and *VvSAP10, VvSAP12* and *VvSAP13,* and *VvSAP14* and *VvSAP15*) are present on chromosome 2, chromosome 6, chromosome 13, chromosome 16 and chromosome 18, respectively. One *SAP* (*VvSAP1, VvSAP11*) is present on chromosome 1 and chromosome 14, respectively.

### 3.3. Multiple Sequence Alignments and Phylogenetic Analysis of SAPs

**Figure 3:**
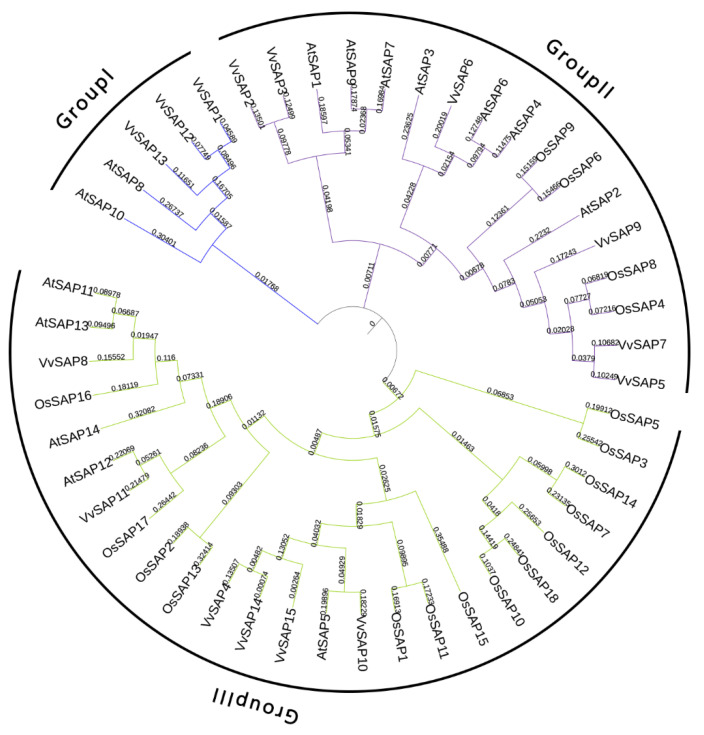
Phylogenetic analysis of SAP proteins from grapevine (*V. vinifera*) and other two plants (*A. thaliana* and *O. sativa*). Sequence alignment was performed with full-length SAP amino acid sequences from different plant species using MAFFT as the default parameters, and the neighbour-joining (NJ) phylogenetic tree was created by MEGA 7.0 using a bootstrap option of 1000 replications.

In order to understand the genetic relationship and evolutionary characteristics of grapevine SAP members, and speculate as to their biological function, an un-rooted phylogenetic tree was constructed by using aligned SAP sequences of grapevine, *Arabidopsis* and rice. As shown in Figure 3, the SAPs from the three species could be divided into three groups, which indicates that the plant SAPs are relatively conservative during evolution. Group I contained two AtSAPs and three VvSAPs. Group II contained seven AtSAPs, six VvSAPs and four OsSAPs. Group III was composed of five AtSAPs, fourteen OsSAPs and six VvSAPs. Group II and Group III included more than half of the total SAPs, ranking the largest grapevine SAPs groups. The grapevine SAPs categorized as Group I members were A20-AN1-type SAPs (VvSAP1 and VvSAP13) and a single AN1 domain (VvSAP12). The grapevine SAPs in group II were almost all the A20-AN1-type (VvSAP2, VvSAP5, VvSAP6, VvSAP7 and VvSAP9), with the only exception of VvSAP3 that contained a single AN1 domain. The grapevine SAPs in group III were A20-AN1-type (VvSAP4, VvSAP8, VvSAP11, VvSAP14 and VvSAP15), with the exception of VvSAP10 which contained a single A20 domain.

### 3.4. Conserved Motif, Typical Domain and Gene Structure Analysis

Gene structure often diverges during the evolution of multigene families. This mechanism of gene evolution facilitates the acquisition of new functions which allow organisms to adapt to various challenging environments. To further investigate the function of *SAP* genes in grapevine, the structural features of *SAPs* were analysed (Figure 1). The N-terminal of *VvSAP1*-*VvSAP10* contained conserved motif one, the C-terminal of *VvSAP1*-*VvSAP13* contained conserved motifs two and three, and the C-terminal of *VvSAP14* and *VvSAP15* contained conserved motifs seven and eight. In addition, *VvSAP3, VvSAP6* and *VvSAP8* also contained conserved motifs five and six.

**Figure 4:**
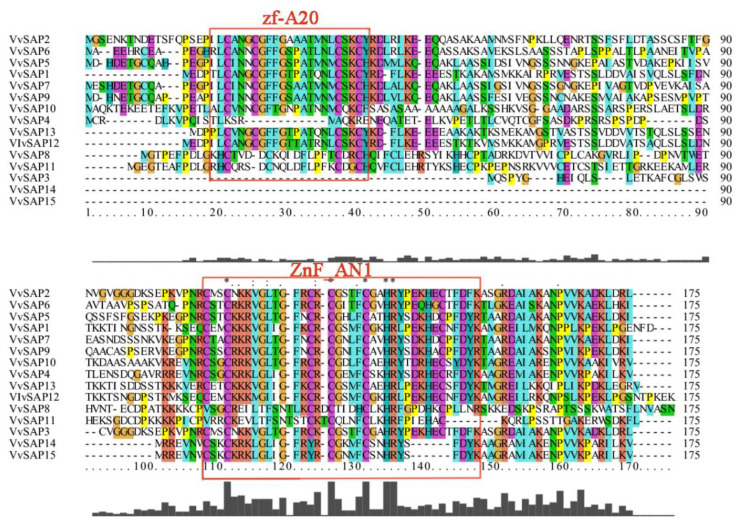
Alignment of conserved domains of SAP family in *V. vinifera*. Alignment of conserved A20/AN1 and grapevine SAP protein sequences. Identical proteins are highlighted in red boxes.

Through the multiple sequence alignment of 15 VvSAP protein sequences (Figure 4), it was found that all of the protein sequences in the SAP family contained the AN1 conserved domain, and most of them contained the zf-A20 conserved domain. Specifically, the VvSAP1-VvSAP15 protein sequences contained the ZnF_AN1 conserved domain, and the VvSAP14 and VvSAP15 protein sequences contained the ZnF_AN1 domain, without the zf_A20 domain. The sequences of this gene family were highly conserved, and they may perform similar functions in grapevine.

### 3.5. Cis-Regulatory Elements in VvSAP Promoters

The sequences 2 kb upstream of the translation initiation site of each *SAP* were analysed using the PLANTCARE server to discover possible elements involved in mediating gene expression. The cis-regulatory elements found in the SAP promoters are shown in Supplementary Material Table S1. Various cis-elements related to plant abiotic stresses, hormone responses and pathogen defences were found in *SAP* promoters. These results indicate that *SAPs* are possibly involved in responses to both biotic and abiotic stresses, and play important roles in plant defence and hormone signalling transduction.

### 3.6. Expression Patterns of VvSAPs in Various Tissues, Organs and Developmental Stages

**Figure 5:**
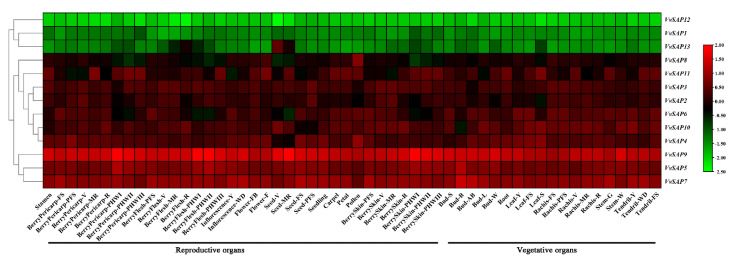
Expression levels of *VvSAP* genes in different tissues of grapevine. Abbreviations stand for plant developmental stages, where FS stands for fruit set, PFS for post-fruit set, V for veraison, MR for mid-ripening, R for ripening, PHWI for post-harvest withering I (1st month), BerryPericarp-PHWII for post-harvest withering II (2nd month) and BerryPericarp-PHWIII for post-harvest withering III (3rd month), Y stands for Young, FB stands for flowering begins, F stands for flowering, ML stands for mature leaf, WD stands for well developed, W stands for woody, S represents different developmental stages in Bud and Leaf, swell in bud and senescence in leaf. Black lines indicate reproductive and vegetative organs, respectively. The colour scale represents log 2 expression values.

The expression patterns of *VvSAPs* in different tissues, organs and developmental stages were analysed (http://bar.utoronto.ca/efp_grape/cgi-bin/efpWeb.cgi, accessed on 15 March 2022. As shown in Figure 5, nearly all *VvSAPs* were up-regulated in both vegetative and reproductive organs, whereas *VvSAP1*, *VvSAP12* and *VvSAP13* were down-regulated. The declined extent was much stronger in vegetative organs than in reproductive organs. Among these genes, *VvSAP9* and *VvSAP7* have the highest transcript levels among all tissues. *VvSAP4* and *VvSAP8* exhibited much higher transcript abundance in pollen than in other flower structures. The consistently down-regulated *VvSAP13* showed an up-regulation in seed and berry. The transcriptome data showed that several *VvSAPs* genes, such as *VvSAP2, VvSAP3, VvSAP4, VvSAP6, VvSAP8* and *VvSAP11*, displayed remarkable transcriptions at certain time points during plant development.

### 3.7. Involvement of VvSAPs in Biotic Stresses

**Figure 6:**
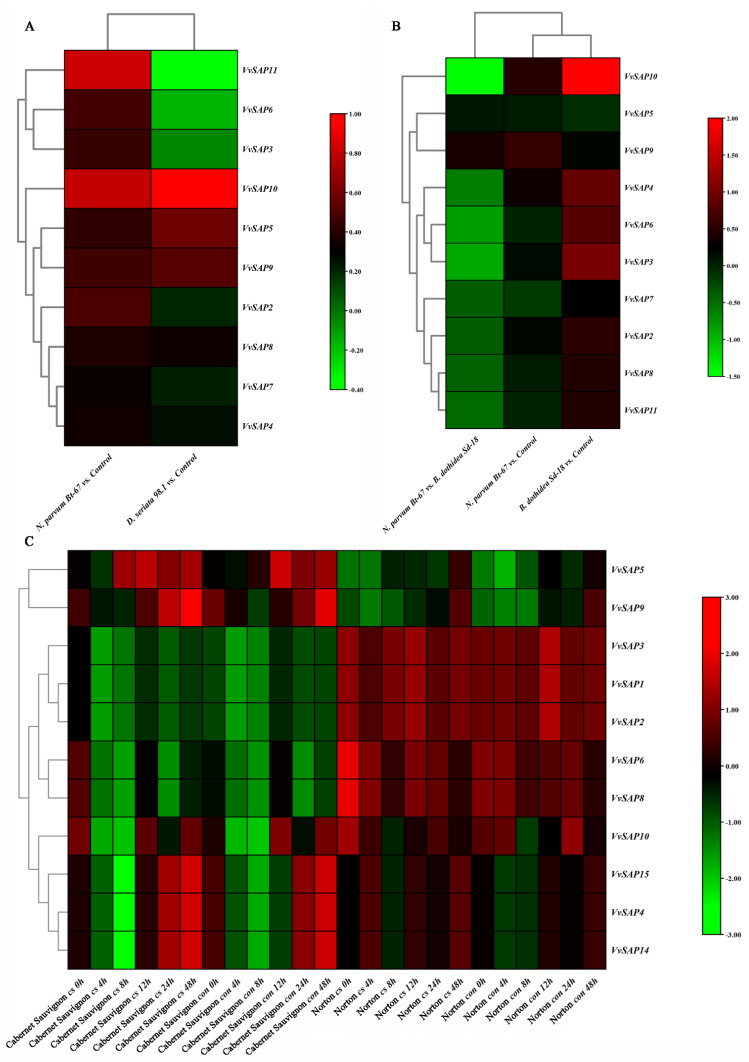
Expression of *VvSAPs* under biotic stresses. *VvSAPs* of *V. rupestris* expressing GFP-*At*TUB6 under the infection of culture filtration of *N. parvum* Bt-67 and *B. dothidea* Sd-18 at 12 hpi (**A**); *N. parvum* Bt-67 and *D. seriata* 98.1 at 3 dpi (**B**); and *VvSAPs* of *V. aestivalis* × *V. vinifera* ‘Norton’ and *V. vinifera* ‘Cabernet Sauvignon’ under infection of conidiospores of *Erysiphe necator* (**C**). cf, culture filtration, cs conidiospores, con, control. The expression level of genes was determined based on the value of FPKM. The colour scale represents log 2 expression values.

To clarify the expression pattern of the grapevine *SAPs* gene family under biotic stress, in this study, the expression levels of the grapevine *SAP* family in native American grapevines (*V. rupestris* expressing GFP-*At*TUB6), native European grapevine (*V. vinifera* ‘Cabernet Sauvignon’) and a hybrid species (*V. aestivalis* × *V. vinifera* ‘Norton’) challenged by fungi infection were compared (Figure 6) [30,33]. *VvSAP10* was shown to be highly induced in *V. rupestris*, expressing GFP-*At*TUB6 inoculated by *N. parvum* Bt-67, *D. seriata* 98.1 and *B. dothidea* Sd-18 at 12 and 72 hpi, respectively (Figure 6A,B). To be specific, the expression levels of *VvSAP10* were more obvious in two less virulent fungi, *D. seriata* 98.1 and *B. dothidea* Sd-18, than in *N. parvum* Bt-67. Moreover, the expression of *VvSAP3, VsSAP6* and *VvSAP11* at 72 hpi were shown to be inverse results between *D. seriata* 98.1 and *N. parvum* Bt-67 inoculation (Figure 6B). Therefore, an earlier (from 0-48 hpi) relatively expression pattern (to control) was shown (Figure 6A). Moreover, the expression levels in tissue of *V. vinifera* ‘Cabernet Sauvignon’ and *V. aestivalis* × *V. vinifera* ‘Norton’ without inoculation and after inoculation with *Erysiphe necator* conidospores were further analysed (Figure 6C). Interestingly, the expression of *VvSAP1*, *VvSAP2* and *VvSAP3* reduced during the early time frame but were resilient nearly to the initial level in both *V. aestivalis* × *V. vinifera* ‘Norton’ and *V. vinifera* ‘Cabernet Sauvignon’, although the relative expression levels were higher in *V. aestivalis* × *V. vinifera* ‘Norton’. The induction of *VvSAP10* presented an earlier induction in *V. vinifera* ‘Cabernet Sauvignon’ (at 12 hpi) than in *V. aestivalis* × *V. vinifera* ‘Norton’ (at 24 hpi). However, the relative expression of *VvSAP15*, *VvSAP4* and *VvSAP14* held a similar pattern to *VvSAP1*, *VvSAP2* and *VvSAP3*, although the final expression levels were higher compared to the initial (Figure 6C).

### 3.8. Involvement of VvSAPs in Abiotic Stresses

**Figure 7:**
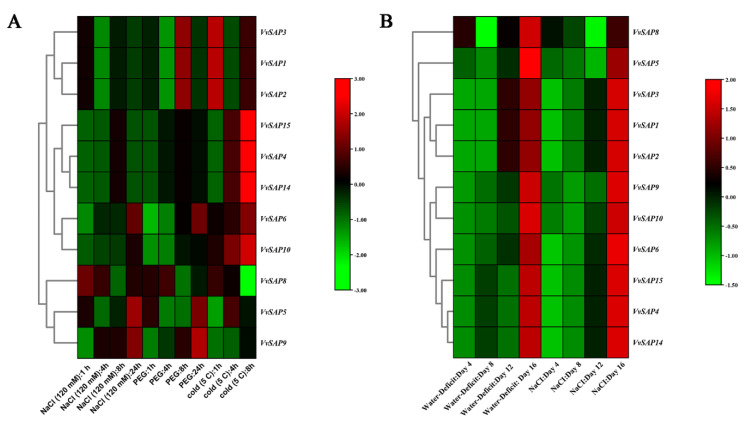
Expression of *VvSAPs* under abiotic stresses. Expression analysis of *VvSAP*s under drought, salt (match water potential to those of the water-deficit-treated plants), and cold (5 ℃) stresses in grapevine in short-term treatment (**A**); and long-term treatment (**B**). The expression level of genes was determined based on the value of RPKM. The colour scale represents log 2 expression values.

With regard to the grapevine distribution evolutionarily, and its cultivation status nowadays, the abiotic stresses that affect grapevine the most are drought, salt and temperature. In this study, the grapevine *SAPs* have been analysed in silico on transcript level based on the GEO database [31,32]. Consistent with plant responses to biotic stress, plant responses to abiotic stress were analysed in both the short time (within 24 h) and the long term (after days), respectively. *VvSAP6* was the most significantly enhanced gene in all of the tested abiotic stresses. Particularly under osmotic stress (PEG 1-24 hpi, Figure 7A, and water deficit 1-16 dpi, Figure 7B), the *VvSAP6* mediated salinity-induced resistance occurred later than osmotic stress (NaCl 1-24 hpi, Figure 7A, and NaCl 4-16 dpi in Figure 7B). The one with obvious expression induced by biotic stress, *VvSAP10*, was attractive to mine its function in abiotic stresses. Contrary to the *VvSAP6*, *VvSAP10* showed mild changes to the osmotic and salinity, but to the temperature stress as promptly as within 8 h (5 °C, Figure 7A). Moreover, *VvSAP9* showed a similar expression pattern to *VvSAP6*, although the extent was not as prominent as the later one (Figure 7A,B). The rest of the *VvSAPs* could be categorized into three groups. *VvSAP15, VvSAP4* and *VvSAP14* were induced continuously from hours to days in all the abiotic stresses. The expression of *VvSAP5* and *VvSAP8* remained fairly steady, with only a bit of down-regulation at day 12 to osmotic and salinity stresses, respectively. *VvSAP1, VvSAP2* and *VvSAP3* were firstly decreased at 4 hpi, but came back to the similar level as initially within 1 d and continued to increase (Figure 7A,B).

## 4. Discussion

Resistance to biotic and abiotic stresses is a central topic for sustainable agriculture, especially in grapevine, one of the world-wide cultivated crops between 30° and 50° N and 30° and 40° S with the highest economic output per acreage. Abiotic stresses strongly affect biotic interactions at various levels, for instance, plant morphogenesis from cells, tissues to organs, transcriptomic regulation, signalling pathways, as well as primary and secondary metabolisms. These factors combine to make an individual plant a more or less suitable host for its counterpart [36,37].

Proteins containing A20/AN1 zinc fingers have been characterised as regulating the immune responses in animals and environmental responses in plants [38]. The grapevine genome database was referred to in this study, and a total of 15 *VvSAP* gene members was identified and characterised (Table 1). Genome-wide survey of *SAP* gene family composition was further performed in other plant species. The family members of *SAPs* in grapevine are more than in maize (11), *Arabidopsis* (14) and tomato (14); while less than in rice (18), potato (17), tobacco (16) and cotton (37). The conservative domain of the *VvSAP* family was analysed (Figure 4). It was found that all candidates contained the conserved AN1 zinc-finger domain and mostly also contained the A20 zinc-finger domain, which is similar to findings in *Arabidopsis*, cotton and tomato, but not in rice. In addition, there were five SAPs in rice and four in *Arabidopsis* with only the AN1 zinc-finger domain. On the other hand, in the evolutionarily primitive organisms such as *Saccharomyces cerevisae* and *Aspergillus fumigatus*, only an AN1 zinc finger domain, being devoid of A20 domain, has been found, and with similar properties in cotton. This may indicate the ancient origin of the AN1 zinc finger domain compared to A20 domain [12,24]. Chromosomal positions of *VvSAP* were identified using Tbtools1.098763 (Figure 2).

Analysis of the evolutionary relationships of SAPs among grapevine and two other species indicated that these SAPs can be divided into three groups. The grapevine SAPs in group II were mostly with the A20-AN1-type, excepting VvSAP3 containing a single AN1 domain (Figure 3). The conserved motif distributions of VvSAP according to the evolutionary relationship were further examined. A total of 10 conserved motifs were identified, and their distributions exhibited strong evolutionary conservation (Figure 1). Gene structure can also effectively reveal evolutionary relationships among gene families. Zhou et al. (2018) reported that the intronless *SAPs* could reduce post-transcriptional processing, and therefore can be utilised to test immediate responses to abiotic stresses [39]. According to these findings, *SAPs* are highly conserved evolutionarily in plants [39]. The chromosomal positions of candidates were also obtained from *V. vinifera* genome annotation: Table 1. The location of *SAP* genes in grapevine can help to identify these genes in other species, directly or using microsynteny among them.

Plant *SAP* genes are thought to play a vital role in regulating a variety of biotic and abiotic stresses according to previous studies [23,40,41]. The most well-characterised plant protein in this class is the rice A20/AN1 zinc-finger domain stress associated protein 1, OsISAP1. Mukhopadhyay et al. (2004) found that the over-expression of *OsISAP1* exhibits tolerances to cold, water defection and salinity stress [19]. Moreover, the *OsSAPs* are responsive to multiple biotic stresses. The overexpression of *OsSAP1* enhances the basal resistance against pathogen infection in tobacco [28]. Our results indicated that nearly all *VvSAP* genes were up-regulated in both vegetative and reproductive organs, whereas *VvSAP1*, *VvSAP12* and *VvSAP13* were down-regulated. The decreased extent was much stronger in vegetative organs than in reproductive organs. *VvSAP4* and *VvSAP8* exhibited a much higher transcript abundance in pollen than in other flower structures (Figure 5). Further, a native American grapevine (*V. rupestris*), a native European grapevine (*V. vinifera* ‘Cabernet Sauvignon’) and a hybrid species (*V. aestivalis* × *V. vinifera* ‘Norton’) were compared respectively for their biotic stress resistance (to Botryosphaeriaceae and Erysiphaceae) (Figure 6). Given that the expression data at the quantitative level were generated by different methods (microarray Figure 5, Figure 6C and Figure 7 vs. RNAseq Figure 6A,B), the comparisons in this study are restricted within the same method. There are significant different expression patterns (nothing to consider their quantitative level) among American and European grapevine varieties. *VvSAP10* was highly induced in *V. rupestris*. The expression levels were more obvious in two less virulent fungi. *D. seriata* 98.1 and *B. dothidea* Sd-18. than in *N. parvum* Bt-67 (Figure 6A,B) [11]. The induction of *VvSAP10* presented an earlier induction in *V. vinifera* ‘Cabernet Sauvignon’ than in *V. aestivalis* × *V. vinifera* ‘Norton’. *VvSAP10* showed mild changes to osmotic and salinity stresses, but strong changes to the temperature stress (Figure 7). *VvSAP6* was the most significantly enhanced gene against all of the tested abiotic stresses. Especially under osmotic stress, the *VvSAP6* mediated salinity-induced resistance occurred later than osmotic stress (Figure 7A,B). The expression pattern of *VvSAP1, VvSAP2, VvSAP3, VvSAP5* and *VvSAP8* was down-regulated in the short term and then enhanced (Figure 7A,B), which is different to the *SAPs* in *Arabidopsis*, cucumber and cotton [20,42].

Temporary water deficit stress could enhance the immunity of *Arabidopsis* by stomatal closure, thereby enhancing the resistance to *Pseudomonas syringae* pv. Tomato DC3000. In this case, a downstream gene transcription level has confirmed that the E3 ligase SINAT4 down-regulated the cysteine protein RD21A [43]. Our previous study on the actin filament (AF) marker line in grapevine indicates that guard cells act as pacemakers of defence, dominating the responses of the remaining epidermal cells. It has been shown that to the phytopathogenic bacteria *P. syringae* pv. Tomato DC3000, actin responses were confined to the guard cells. In contrast, upon contact with zoospores of *Plasmopara viticola*, not only the guard cells, but also epidermal pavement cells where no zoospores had attached, responded with the formation of a perinuclear actin basket [44]. 

The cold stress normally inhibits the JA-dependent signalling pathway but activates the salicylic acid (SA)-dependent signalling pathway; the high temperature brings a contrary consequence [36]. Although the gene expression levels induced by abiotic stresses are normally much milder than the biotic stress induction (Figure 6 and Figure 7), it probably can affect the activation of certain resistance genes in plants challenged by further attacks of pathogenic microorganisms [36]. Paolinelli-Alfonso et al. (2016) found that transcriptome analysis of *L. theodromae* in the presence of grapevine wood revealed an upregulation of genes involved in the SA pathway [45]. This upregulation was enhanced by high temperature. The high temperature-induced contradictory SA signalling pathway can be understand by taking account of the biotic induced signalling. An activation of L-tyrosine catabolism pathway could lead to inhibition of the SA pathway, favouring fungal development especially during heat stress [45]. 

On the other hand, high JA concentration is known to have an antagonistic effect on the SA pathway [46]. Botryosphaeriaceae are known to produce JA, which has been identified in the culture filtrate of some GTD agents, e.g., *N. parvum* and *N. vitifusiforme* [47]. Hence it could be hypothesised that, in addition to a toxic effect, JA inhibits the SA pathway involved in the stimulation of plant defences. Zhao et al. (2021) have reported a downregulation of *VvWRKY70* [30], a putative central component of the SA pathway, after treatment of *V. rupestris* cells with *D. seriata* secreted compounds.

These signalling cascades are partially sharing with abiotic induced defence responses, although it is to be noted that this is a matter of timing events. Otherwise, a consequence-centred explanation may lead to an apparent discrepancy. For instance, under cold tolerance study, a transient disassembly of microtubules followed by their recovery with progressive acclimation has been approved to be a kind of cold acclimation, which is obviously contradictory if to follow the final consequence that a stable microtubules array was established for cold challenging [48]. Moreover, the above discussed JA is necessary to convey efficient disassembly of MTs under cold stress, but that is not the exclusive signal. Another example comes from the cytoskeleton (actin filaments and microtubules) early arrangement (within a time frame of 20 min vs. long-time adaptation, e.g., hours or days) to distinguish the PTI from ETI [49]. This evidence could come back to answer the basic question, that environmental mediated biotic stress symptoms are not always coincidental with the invasion of casual agents.

The above findings indicate crosstalk and convergence of mechanisms in these pathways and the existence of a general stress response, suggesting that resource allocation could be subjected to a trade-off between vessel occlusion and defence reaction in conjunctive living tissues in grapevine, as well as the signalling transduction cascade within single cells and amongst the symplasts through plasmodesmata. The principle of the CODIT lies in the compartmentalisation of the established four obstacles that restrict and decay pathogen spread [50]. The first three obstacles located in xylem per se can be interpreted as the reaction zone after pathogen invasion. Obstacle four, consisting of living cells, e.g., paratracheal parenchyma, fibres and ray, can protect the host via arming themselves with newly formed structures. The mystery of environment-related grapevine disease, such as the disruption of water flow, can probably be explained via vessel dependence systemically spread, as well as local living cell signalling transduction against these four obstacles [10]. VaSAP15 from a Chinese wild grapevine *V. amurensis* has been confirmed to localise in both the nucleus and cell membrane, which prompts a pending question during the penetration of fungi, that the contraction of the actin around the penetration site probably linked with the actin nucleus basket to attract the nucleus towards the penetration site [29,51]. Plant-specific class XIV kinesin KCH1 has been confirmed for its function as a tether [9,52]. Therefore, the *VvSAPs* are probably triggered at the early time point via an acting-dependent endocytotic of a receptor on the member.

## 5. Conclusions

In the present study, the systematic identification and characterisation of *SAP* gene family members in grapevine were studied. Their characteristics, including genomic locations, gene duplications, evolutionary relationships, conserved domains and motifs, gene structures and cis-elements in promoter regions were analysed based on bioinformatics methods. In addition, the regulations of *VvSAP*s in biotic and abiotic stresses were investigated based on transcriptomic analysis and discussed under the frame of signalling cross-talking at cellular and organic level. On the one hand, since plant response to abiotic stress must be coordinated with growth and development, such a host physiological status further confers susceptibility or resistance through biotic/abiotic signalling cross-talking under biotic stress; on the other hand, endophytic and rhizospheric microorganisms can enhance plant resistance to abiotic stresses, and understanding the biotic/abiotic signalling cross-talking can be helpful for the utilisation of biocontrol. This study provides essential information for elucidating the potential roles of *VvSAP* in response to biotic and abiotic stresses. Further studies focus on the respective roles of the different *SAPs* investigated via transgenic overexpression or knock-outs and can provide direct evidences and potential implications for grapevine breeding.

## Figures and Tables

**Table 1:** Identification and characterisation of SAP family genes in grapevine.

Gene Name.	Accession Number	Protein	MW	pI	GRAVY	Aliphatic Index	Loc	Chrom
***VvSAP1***	VIT_01s0011 g02290.t01	154	17280.02	8.78	−0.661	52.6	Nucleus	Chr1
***VvSAP2***	VIT_02s0025 g05050.t01	172	18460.83	8.77	−0.494	46.05	Nucleus	Chr2
***VvSAP3***	VIT_02s0025 g05080.t01	100	10918.54	8.98	−0.498	52.7	Nucleus	Chr2
***VvSAP4***	VIT_06s0004 g01790.t01	142	16150.55	9.37	−0.849	57.68	Nucleus	Chr6
***VvSAP5***	VIT_06s0004 g01820.t01	172	18502.95	7.99	−0.5	56.8	Nucleus	Chr6
***VvSAP6***	VIT_08s0007 g03530.t01	161	17192.68	8.92	−0.3	66.21	Nucleus	Chr8
***VvSAP7***	VIT_08s0007 g07950.t01	172	18153.57	7.99	−0.327	68.72	Nucleus	Chr8
***VvSAP8***	VIT_08s0007 g08360.t01	293	32457.78	8.56	−0.614	58.84	Nucleus	Chr8
***VvSAP9***	VIT_13s0064 g01210.t01	172	18504.11	8.22	−0.371	61.4	Nucleus	Chr13
***VvSAP10***	VIT_13s0064 g01220.t01	172	18359.69	8.95	−0.486	57.5	Nucleus	Chr13
***VvSAP11***	VIT_14s0066 g01880.t01	189	21133.11	8.74	−0.8	47.99	Nucleus	Chr14
***VvSAP12***	VIT_16s0022 g01680.t01	239	26461.39	8.98	−0.772	51.46	Chloro-plast Nucleus	Chr16
***VvSAP13***	VIT_16s0022 g01980.t01	152	16850.54	8.77	−0.558	58.36	Nucleus	Chr16
***VvSAP14***	VIT_18s0001 g00430.t01	67	7980.5	10.26	−0.676	59.7	Nucleus	Chr18
***VvSAP15***	VIT_18s0001 g01260.t01	67	7966.43	10.2	−0.67	59.7	Nucleus	Chr18

## Data Availability

Not applicable.

## Supplementary Materials

The following supporting information can be downloaded at: https://www.mdpi.com/article/10.3390/pathogens11121426/s1, Table S1: The cis-elements in the *VvSAP* promoters.
